# Retraction: Identification of early diagnostic and prognostic biomarkers via WGCNA in stomach adenocarcinoma

**DOI:** 10.3389/fonc.2024.1459966

**Published:** 2024-07-16

**Authors:** Frontiers Editorial Office

The journal retracts the 18 June 2021 article cited above.

Following publication, concerns were raised regarding the integrity of the images in the published figures. The authors failed to provide a satisfactory explanation during the investigation, which was conducted in accordance with Frontiers’ policies. As a result, the data and conclusions of the article have been deemed unreliable and the article has been retracted.

This retraction was approved by the Chief Editors of Frontiers in Oncology and the Chief Executive Editor of Frontiers. The authors received a communication regarding the retraction and had a chance to respond. This communication has been recorded by the publisher.

